# Effects of Metal Ions, Temperature, and a Denaturant on the Oxidative Folding Pathways of Bovine α-Lactalbumin

**DOI:** 10.3390/ijms18091996

**Published:** 2017-09-16

**Authors:** Reina Shinozaki, Michio Iwaoka

**Affiliations:** Department of Chemistry, School of Science, Tokai University, Kitakaname, Hiratsuka-shi, Kanagawa 259-1292, Japan; brekekekex.koax.koax49@gmail.com

**Keywords:** lactalbumin, protein folding, folding intermediate, disulfide bond, oxidation, reduction, calcium binding site, metal ion, temperature effect, selenoxide

## Abstract

Bovine α-lactalbumin (αLA) has four disulfide (SS) bonds in the native form (N). On the oxidative folding pathways of this protein, two specific SS folding intermediates, i.e., (61–77, 73–91) and des[6–120], which have two and three native SS bonds, respectively, accumulate predominantly in the presence of Ca^2+^. In this study, we reinvestigated the pathways using a water-soluble cyclic selenoxide reagent, *trans*-3,4-dihydroxyselenolane oxide (DHS^ox^), as a strong and quantitative oxidant to oxidize the fully reduced form (R). In the presence of ethylenediaminetetraacetic acid (EDTA) (under a metal-free condition), SS formation randomly proceeded, and N did not regenerate. On the other hand, two specific SS intermediates transiently generated in the presence of Ca^2+^. These intermediates could be assigned to (61–77, 73–91) and des[6–120] having two common SS bonds, i.e., Cys61-Cys77 and Cys73-Cys91, near the calcium binding pocket of the β-sheet domain. Much faster folding to N was observed in the presence of Mn^2+^, whereas Na^+^, K^+^, Mg^2+^, and Zn^2+^ did not affect the pathways. The two key intermediates were susceptible to temperature and a denaturant. The oxidative folding pathways revealed were significantly different from those of hen egg white lysozyme, which has the same SS-bonding pattern as αLA, suggesting that the folding pathways of SS-containing proteins can alter depending on the amino acid sequence and other factors, even when the SS-bond topologies are similar to each other.

## 1. Introduction

Folding of disulfide (SS)-rich proteins proceeds via sequential formation of the SS bonds between two cysteine (Cys) residues. This folding process involves two chemical events: random SS formation to generate a mixture of various SS intermediates and SS rearrangement to produce distinct SS intermediates having partially native-like fold [1,2]. In vivo, the oxidative folding of a protein is catalyzed by various redox enzymes, such as protein disulfide isomerases [3,4,5], which effectively promote both oxidation (SS formation) and isomerization (SS rearrangement) in the endoplasmic reticulum to guide a nascent polypeptide chain to the native protein. In vitro, on the other hand, redox buffers, such as reduced glutathione (GSH)/oxidized glutathione (GSSG) and dithiothreitol (DTT^red^)/*trans*-4,5-dihydroxy-1,2-dithiane (DTT^ox^), are usually applied to accelerate the folding events. Many SS-containing proteins have already been investigated for their oxidative folding pathways under neutral to slightly basic conditions [6,7,8]. According to the previous studies, the folding pathways can be classified between two extreme cases [9]. One extreme is the folding of hirudin having three SS bonds, for which no distinct SS intermediate is generated and, instead, hundreds of SS intermediates that have statistically formed SS bonds appear in the folding solution [10,11]. In the folding of this type, a native form (N) slowly generates by trial-and-error SS reshuffling. The other extreme is the folding of bovine pancreatic trypsin inhibitor (BPTI) having three SS bonds, for which only distinct SS intermediates with partially native-like fold sequentially generate from the fully reduced form (R) to N [12]. For other proteins, such as bovine pancreatic ribonuclease A (RNase A) and hen egg white lysozyme (HEL), the folding proceeds through the pathways lying between these two extreme cases [13,14,15,16]. Incorrect protein folding (misfolding) may lead to the formation of protein deposits, inclusions, fibrils, etc., associated with various human diseases [17].

Bovine milk α-lactalbumin (αLA), which has four SS bonds, i.e., Cys6-Cys120, Cys28-Cys111, Cys61-Cys77, and Cys73-Cys91 [18], is a representative whey protein and has attracted an interest not only from a viewpoint of protein folding [19,20,21,22,23,24,25,26,27], but also in relation to its immune modulation and anti-carcinogenic activities [28]. Regarding the former point, Kuwajima and coworkers recently reported interesting folding behaviors of goat α-lactalbumin and homologous canine milk lysozyme: although these proteins shared similar backbone topology, the folding pathways of the SS-intact proteins were different [29]. As to the latter concern, the specific complex formed between partially unfolded apo-αLA in a molten globule-like conformation [30] and unsaturated C18 fatty acids was suggested to play a key role [31,32].

In the meantime, it was found by the SS-coupled folding study that αLA exhibits different behaviors in the oxidative folding pathways depending on the presence or absence of Ca^2+^ [33,34,35,36]. In the absence of Ca^2+^, the folding was hirudin-like, forming ensembles of randomly formed SS intermediates with one to four SS bonds, i.e., 1SS, 2SS, 3SS, and 4SS, respectively. On the other hand, in the presence of Ca^2+^, αLA folded through BPTI-like folding pathways, in which unique 2SS and 3SS intermediates were predominantly accumulated. Chang [34,35] performed very precise analysis of the SS intermediates observed during the oxidative folding of αLA at pH 8.4 and 22 °C and successfully specified the SS bonds in the two unique SS intermediates: the one was (61–77, 73–91) having two native SS bonds, and the other was des[6–120] having three native SS bonds but lacking the native Cys6-Cys120 SS bond. Note that these intermediates were called αLA-IIA and αLA-IIIA, respectively, in previous studies [35,36]. Thus, it was suggested that among four native SS bonds Cys61-Cys77 and Cys73-Cys91, which are located near the calcium binding pocket of the β-sheet domain, predominantly form in the presence of Ca^2+^ [22]. These intermediates could be converted to N by oxidation. However, as to the effects of temperature, a denaturant, pH, and coexisting metal ions (other than Ca^2+^) on the oxidative folding pathways of αLA, the details have not been elucidated to date, in spite of extensive studies having been done for αLA with the SS bonds intact (for recent studies, see [29,37]). This was because the oxidants previously available for oxidative folding study, such as GSSG and DTT^ox^, had low oxidation potentials, thereby restricting the applicable folding conditions in a narrow range.

We recently developed a new oxidant, i.e., *trans*-3,4-dihydroxyselenolane oxide (DHS^ox^), which is a water-soluble cyclic selenoxide reagent having a high oxidation potential (i.e., a strong oxidant) and is capable of rapid and stoichiometric SS formation of proteins [38,39]. By using this reagent, oxidative folding pathways of RNase A, hirudin, and HEL were investigated in a wide range of folding conditions [39,40,41,42]. For example, when it was applied to HEL [42], which has four native SS bonds of Cys6-Cys127, Cys30-Cys115, Cys64-Cys80, and Cys76-Cys94, three des intermediates (i.e., des[76–94], des[64–80], and des[6–127]) were clearly observed at pH 8.0 and 5 °C. Native HEL was predominantly regenerated by oxidation of des[64–80] and des[6–127] at temperatures below 25 °C, being consistent with the literature [16]. However, at 35 °C, des[76–94] was only a possible precursor to native HEL. Thus, it was found that HEL switches the major folding pathways depending on the temperature [42].

**Figure 1:**
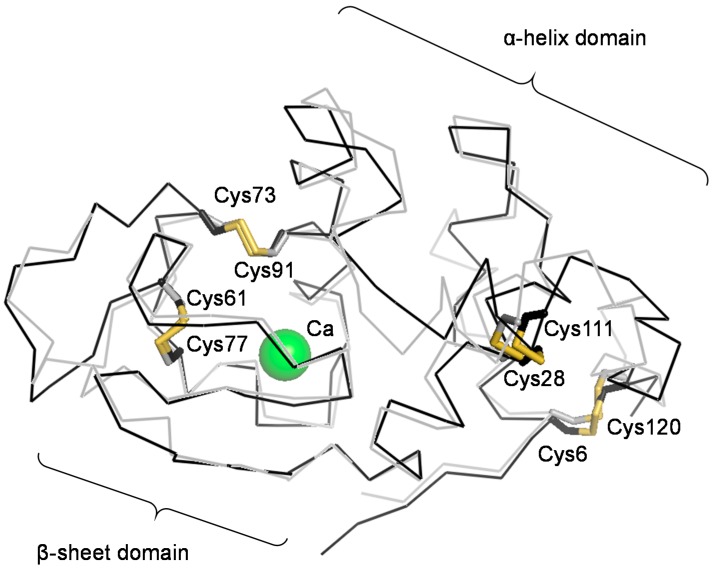
Molecular structure of bovine milk αLA (black, pdbcode: 1hfz) superimposed onto the structure of HEL (gray, pdbcode: 6lyz). Locations of Ca^2+^ (green) and SS bonds (yellow) of αLA are shown.

Since HEL and αLA have the same SS-bonding pattern (Figure 1) [43], it is an interesting issue to compare the oxidative folding pathways of these two homologous proteins under similar folding conditions. We, therefore, reinvestigated here the oxidative folding pathways of αLA by using DHS^ox^ in the absence or presence of various metal ions. The effects of temperature and a denaturant on the folding pathways were also investigated.

## 2. Results

### 2.1. Stoichiometric Oxidation of R Using DHS^ox^

**Figure 2:**
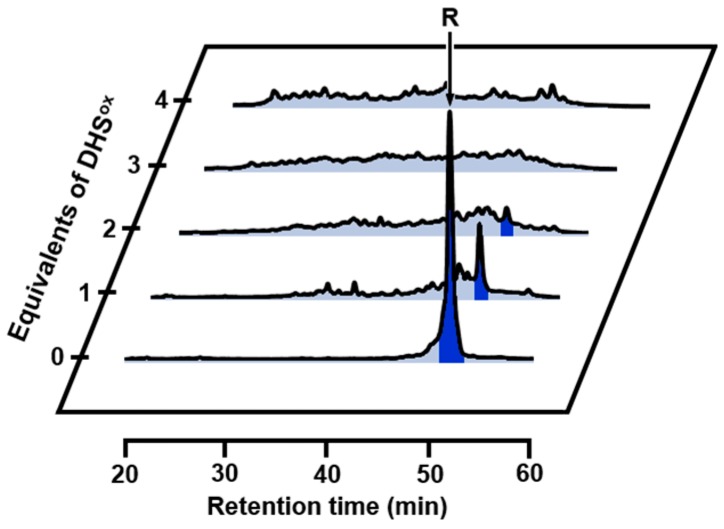
Reverse-phase high performance liquid chromatography (RP-HPLC) chromatograms obtained by the oxidation of R (10 µM) with 1–4 eq *trans*-3,4-dihydroxyselenolane oxide (DHS^ox^) at pH 8.0 and 5 °C in the presence of 10 mM ethylenediaminetetraacetic acid (EDTA). The reaction was acid-quenched after 1 min. For HPLC analysis conditions, see the experimental section.

When R was reacted with 1 eq DHS^ox^ for 1 min at pH 8.0 and 5 °C in the presence of 2 mM ethylenediaminetetraacetic acid (EDTA) (under a metal-free condition), an ensemble of 1SS intermediates formed as a main product accompanied by small amounts of R and an ensemble of 2SS intermediates as revealed for 2-aminoethyl methanethiosulfonate (AEMTS)-quenched sample solution by electrospray ionization-mass spectrometry (ESI-MS) analysis (see Figure S1 and Table S1). AEMTS rapidly reacts a cysteinyl thiol (SH) group to modify it to -SSCH_2_CH_2_NH_3_^+^ without affecting conformations of the folding intermediates [13]. Thus, the AEMTS-quenched intermediates should have a larger mass than N depending on the number of SH groups present in the peptide chain. Similarly, 2SS, 3SS, and 4SS intermediate ensembles were obtained as major products when R was reacted with 2, 3, or 4 eq DHS^ox^, respectively. The results clearly demonstrated that DHS^ox^ rapidly and quantitatively reacted SH groups of R to make SS linkages in the folding intermediates. Reverse-phase high performance liquid chromatography (RP-HPLC) analysis of the acid-quenched samples indicated that the SS formation took place randomly to produce non-specific 1SS to 4SS intermediates (Figure 2). This was consistent with the previous observation that SS formation proceeds more rapidly than SS rearrangement by using DHS^ox^ [39].

### 2.2. SS Rearrangement of the Folding Intermediates

**Figure 3:**
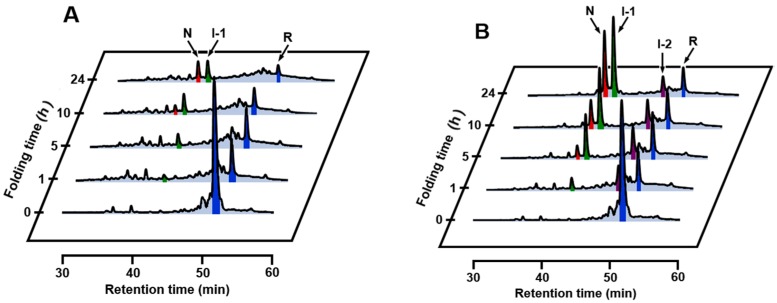
RP-HPLC chromatograms obtained by the oxidation of R (10 µM) with 1 eq DHS^ox^ at pH 8.0 and 5 °C in the absence of EDTA. The reaction was acid-quenched after 0, 1, 5, 10, and 24 h. For HPLC analysis conditions, see the experimental section: (**A**) in the absence of CaCl_2_; (**B**) in the presence of 5 mM CaCl_2_.

**Figure 4:**
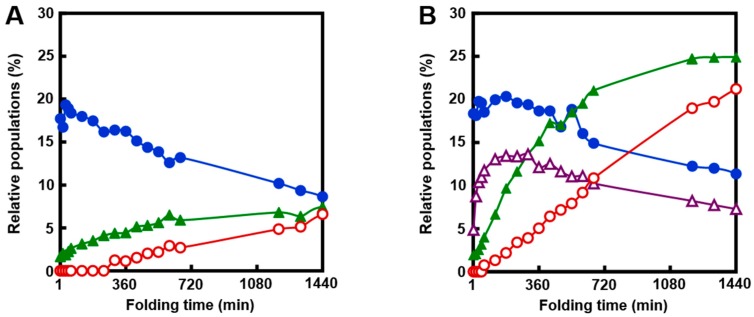
Relative populations of R (●, blue), N (○, red), I-1 (▲, green), and I-2 (△, purple) as a function of a folding time; (**A**) in the absence of CaCl_2_; (**B**) in the presence of 5 mM CaCl_2_. Reaction conditions were the same as Figure 3.

To investigate whether the folding intermediates of αLA initially formed by the reaction of R with DHS^ox^ could transform to specific SS intermediates through SS rearrangement, the reaction was acid-quenched after a long reaction time. However, the HPLC chromatograms (Figure 2) remained unchanged for 24 h in the presence of EDTA (under a metal-free condition). On the other hand, when the experiment was carried out with 1 eq DHS^ox^ in the absence of EDTA, formation of one specific SS intermediate (I-1) was clearly observed with a slight amount of N after 5 h (Figure 3A). It is of note that formation of I-1 was remarkably enhanced and another SS intermediate (I-2) also generated when the same folding was carried out in the presence of 5 mM CaCl_2_ (Figure 3B). Figure 4 shows the changes of relative populations of R, N, I-1 and I-2 as a function of a folding time. In the absence of CaCl_2_, the relative population of R gradually decreased, while N generated slowly. These population changes occurred probably because a trace amount of metal ions remaining in the solution catalyzed air oxidation. Since N and I-1 did not accumulate in the presence of EDTA (Figure 2), presence of some metal ions, which can be captured by EDTA, should be requisite for the formation. In the presence of CaCl_2_, oxidation of R took place at a similar or a little slower reaction rate than that in the absence of CaCl_2_ (Figure 4B). However, generation of N was much faster than that in the absence of CaCl_2_. Moreover, the relative population of I-1 was greater, and a new SS-intermediate I-2 formed earlier than formation of I-1. N started to form after accumulation of I-1. These observations suggest that I-2 is a precursor of I-1 and I-1 is a precursor of N on the folding pathways. Thus, it is clear that a calcium ion (Ca^2+^) enhances the formation of I-1, I-2, and N, although it cannot accelerate air oxidation.

### 2.3. Effects of Metal Ions

**Figure 5:**
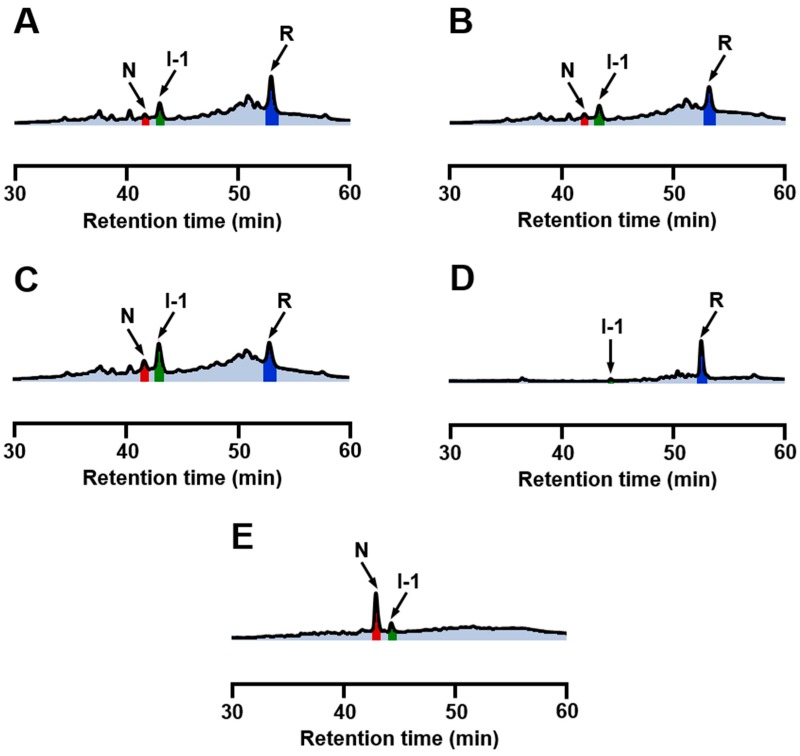
RP-HPLC chromatograms obtained by the oxidation of R (10 µM) with 1 eq DHS^ox^ at 5 °C in the presence of various metal ions. For HPLC analysis conditions, see the experimental section. (**A**) in the presence of 5 mM NaCl at pH 8.0 after 10 h; (**B**) in the presence of 5 mM KCl at pH 8.0 after 10 h; (**C**) in the presence of 5 mM MgCl_2_ at pH 8.0 after 10 h; (**D**) in the presence of 5 mM ZnCl_2_ at pH 6.8 (0.1 M Bis-Tris buffer) after 20 h; and (**E**) in the presence of 0.5 mM MnCl_2_ at pH 8.0 (60 mM Tris buffer) after 5 h.

It is known that metal ions, such as Mg^2+^ and Na^+^, can be weakly bound to the Ca^2+^ binding site existing in the β-sheet domain of αLA when Ca^2+^ is depleted [44,45,46]. Thus, oxidative folding experiments were carried out at pH 8.0 and 5 °C in the presence of various metal chlorides instead of CaCl_2_. The concentrations of NaCl, KCl, MgCl_2_, and ZnCl_2_ were 5 mM except for MnCl_2_, for which it was set to 0.5 mM because the solution color changed dark probably due to decomposition of manganese(II) hydroxide at the concentration of 5 mM. In the case of ZnCl_2_, since the precipitation of zinc hydroxide occurred at pH 8.0, the oxidative folding was carried out at pH 6.8 and 5 °C (see Figure S2 for oxidative folding of αLA at pH 6.8 and 5 °C in the absence and presence of CaCl_2_). The HPLC chromatograms obtained in the presence of NaCl, KCl, and MgCl_2_ (Figure 5A–C) were in accordance with those obtained in the absence of both CaCl_2_ and EDTA (Figure 3A), suggesting that the oxidative folding pathways are quite similar to each other, although one may notice that the formation of I-1 is slightly enhanced by MgCl_2_. Thus Na^+^, K^+^, and Mg^2+^ would not coordinate to the Ca^2+^ binding site of αLA practically in the reduced unfolded state, thereby affecting not at all the thermodynamic stability of the early SS folding intermediates. Indeed, the association constants reported for αLA with these metal ions (100 ± 10, 8 ± 3, and 2000 ± 100 M^−1^, respectively) were much smaller than that with Ca^2+^ (3 × 10^8^ M^−1^) at 20 °C [46]. For the case of Zn^2+^, for which αLA has different binding sites from that for Ca^2+^, only a little amount of I-1 was generated even after 20 h (Figure 5D). This was because the reaction was carried out at pH 6.8, under which condition SS rearrangement and air oxidation of the SS intermediates should be significantly slow. In the presence of Mn^2+^, on the other hand, only a small amount of I-1 was observed, and instead N already generated dominantly after 5 h, even though the concentration of MnCl_2_ was 0.5 mM (Figure 5E). Thus, the oxidative folding proceeded more rapidly than in the presence of Ca^2+^. The reason why Mn^2+^ can effectively guide the SS intermediates to N rather than to misfolded 4SS intermediates is not clear at this moment, but the following features of Mn^2+^ would contribute to the observed strong bias to N. Firstly, the association constant of Mn^2+^ to αLA is as large as that of Ca^2+^ [46]. The strong binding to the Ca^2+^ binding site would enhance thermodynamic stability of I-1 to a significant extent. Secondly, Mn^2+^ would catalyze air oxidation of Cys SH groups to facilitate SS formation due to the variable oxidation state of a manganese atom.

### 2.4. Identification of I-1 and I-2

The two specific SS intermediates observed during the oxidative regeneration of αLA, i.e., I-1 and I-2, would be key intermediates on the folding pathways to N. To characterize the SS-bonding structures of these intermediates, they were isolated and quenched by AEMTS. The ESI-MS analysis revealed that the molecular masses were 14,328.7 Da for I-1 and 14,482.9 Da for I-2 (see Figure S3 and Table S2), indicating that they have two and four AEMTS units (i.e., -SCH_2_CH_2_NH_3_^+^) attached, respectively. The results confirmed that I-1 is a 3SS species and I-2 is a 2SS species. Structures of I-1 and I-2 could be identified to be des[6–120] and (61–77, 73–91), respectively, by comparing the observed HPLC retention times with those reported in the literature [35,36]. It is interesting to note that both I-1 and I-2 have two native SS bonds, i.e., Cys61-Cys77 and Cys73-Cys91, near the calcium binding site of αLA [35]. Complexation of Ca^2+^ should enhance thermodynamic stability of the β-sheet domain and allow the two native SS bonds to form smoothly. This would be the reason for I-1 and I-2 hardly being observed in the absence of Ca^2+^.

### 2.5. Structural Stability of N, I-1, and I-2

**Figure 6:**
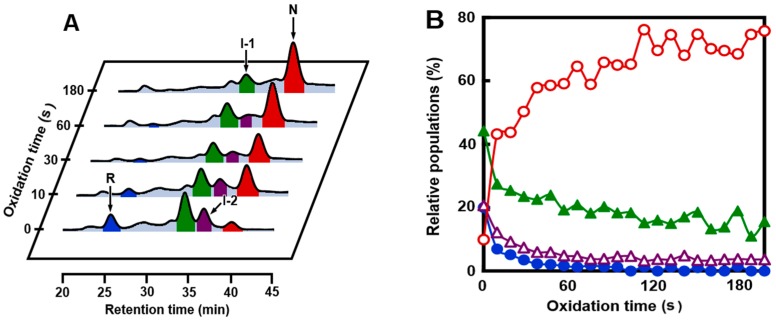
Oxidation pulse experiments. (**A**) RP-HPLC chromatograms obtained 0 to 180 s after addition of 1 eq DHS^ox^ to the reaction mixture, which was obtained by the reaction of R and 1 eq DHS^ox^ at pH 8.0 and 5 °C after 5 h. The oxidative folding conditions were the same as those of Figure 3B, but the reaction was quenched by AEMTS; (**B**) relative populations of R (●, blue), N (○, red), I-1 (▲, green), and I-2 (△, purple) as a function of the oxidation time.

When R was reacted with 1 eq DHS^ox^ in the presence of Ca^2+^, I-2 was accumulated maximal after 5 h (Figure 3B and Figure 4B). At this stage, one more equivalent of DHS^ox^ was added to the reaction mixture. This oxidation pulse should oxidize R and SS intermediates rapidly, since DHS^ox^ is a strong and quantitative oxidant for Cys SH groups. Figure 6A shows the RP-HPLC chromatograms obtained after the oxidation pulse for the sample solutions quenched by AEMTS. It is seen that R disappeared rapidly, whereas oxidation of I-2 proceeded slowly (within 1 min). I-1 remained unoxidized even after three minutes. The consumed amounts of I-1 and I-2 were almost compensated by the produced amount of N (Figure 6B). The results supported that I-1 and I-2 have native SS bonds and are key intermediates on the folding pathways toward N.

**Figure 7:**
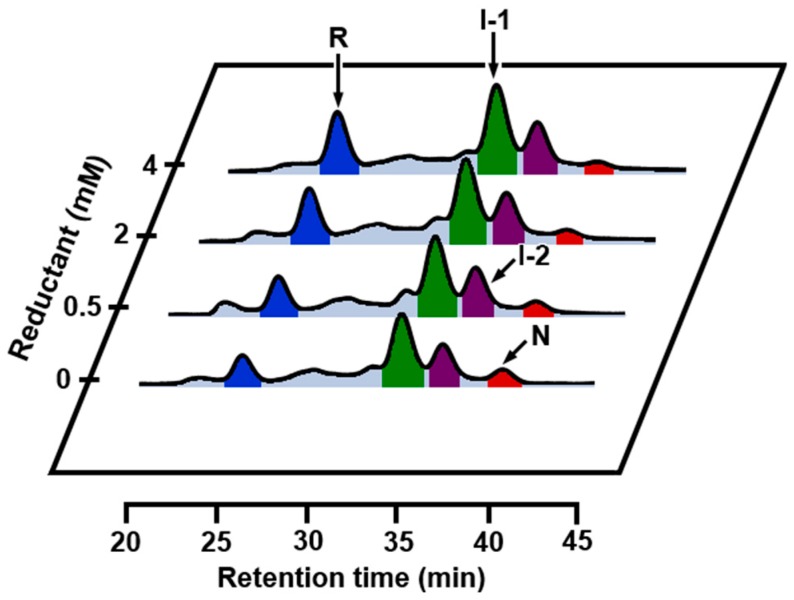
Reduction pulse experiments. RP-HPLC chromatograms obtained three minutes after addition of indicated concentrations of DTT^red^ to the reaction mixture, which was obtained by the reaction of R and 1 eq DHS^ox^ at pH 8.0 and 5 °C after 5 h. The oxidative folding conditions were the same as those of Figure 3B, but the reaction was quenched by AEMTS.

On the other hand, when the same sample solution as that utilized in the above oxidation pulse experiment was added with various amounts of DTT^red^ (Figure 7), reduction of the peak area of N was observed, while the peak area of I-2 did not change and those of I-1 and R were increased. The increased population of R can be explained by the reduction of random SS intermediates by DTT^red^. On the other hand, the enhanced peak area of I-1 would be due to the reduction of the Cys6–Cys120 SS bond in N. This native SS bond is present in the α-domain and exposed outward, so it is expected to react with DTT^red^ first among the four native SS bonds [47,48]. In the meantime, I-1 and I-2, which have two native SS bonds near the calcium binding site, were not reduced by DTT^red^ probably because the Ca^2+^ binding would kinetically stabilize the local folded structure significantly.

### 2.6. Effects of Temperature and a Denaturant

**Figure 8:**
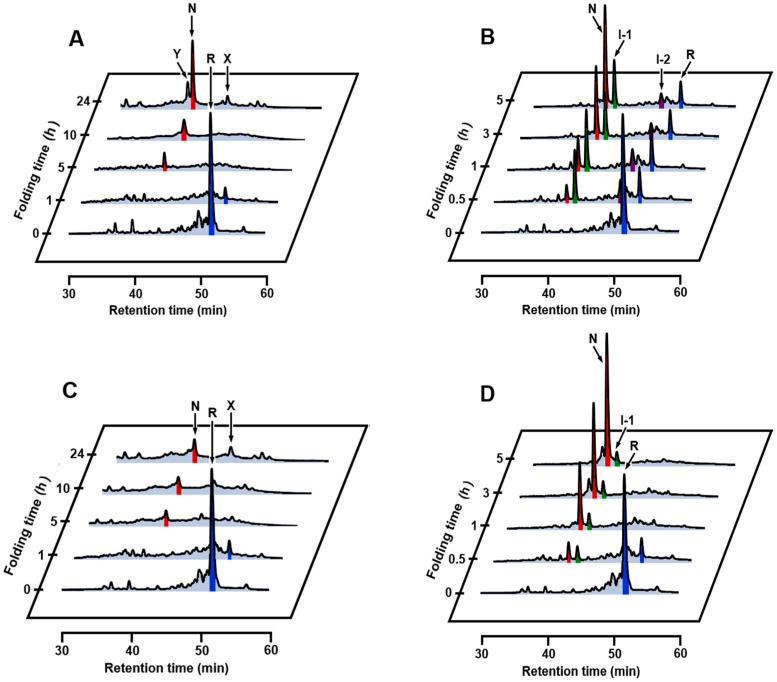
RP-HPLC chromatograms obtained by the oxidative folding of αLA using 1 eq DHS^ox^ at pH 8.0 and 35 or 45 °C in the absence or presence of CaCl_2_. The initial concentration of R was 10 µM. The folding intermediates were trapped at different reaction times by acidification. (**A**) at 35 °C in the absence of CaCl_2_; (**B**) at 35 °C in the presence of 5 mM CaCl_2_; (**C**) at 45 °C in the absence of CaCl_2_; (**D**) at 45 °C in the presence of 5 mM CaCl_2_.

The results of oxidative folding of αLA at 35 °C in the absence and presence of Ca^2+^ are shown in Figure 8A,B, respectively. Both in the absence and presence of Ca^2+^, the oxidative folding progressed more rapidly than that at 5 °C, but the intermediates observed were distinctly different. In the absence of Ca^2+^, the specific SS intermediates, i.e., I-1 and I-2 were not observed, but N already started to form after 3 h. After 24 h, formation of two distinct peaks (X and Y) were observed. These would be misfolded 4SS intermediates, which were previously denoted as X-αLA [49,50]. In the presence of Ca^2+^, I-2 initially formed and then I-1 and N sequentially generated as observed at 5 °C. Comparison of the folding intermediates observed at 5 °C with those observed at 35 °C suggested that I-2 is more susceptible to heat denaturation than I-1. The reason for the more rapid formation of N at 35 °C is ascribed to accelerated air oxidation (SS formation) and SS rearrangement by elevated temperature. The folding was also carried out at 45 °C (Figure 8C,D). Generation of I-2 and I-1 was depressed even in the presence of Ca^2+^, but the folding yield of N already reached to 44% in 5 h. In contrast, in the absence of Ca^2+^, a much less amount of N was generated. This would be due to decreased thermodynamic stability of N free of Ca^2+^. Indeed, thermal denaturation curves (see Figure S4) showed that αLA is less affected in the presence of Ca^2+^ by heat than that in the absence of Ca^2+^. The HPLC chromatogram of scrambled 4SS intermediates generated by the reaction with 1 eq DHS^ox^ in the absence of Ca^2+^ at 45 °C (Figure 8C, 24 h) was roughly similar to the one obtained at 5 °C in the presence of EDTA (Figure 2, 4 eq DHS^ox^). Thus, on the oxidative folding pathways of αLA by using DHS^ox^ as an oxidant, formation of I-1 should be essential to efficient regeneration of N.

**Figure 9:**
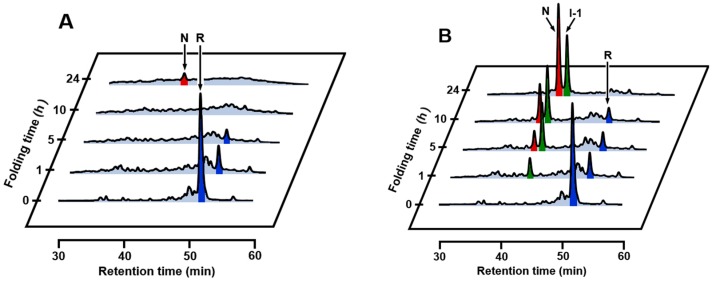
RP-HPLC chromatograms obtained by the oxidative folding of αLA using 1 eq DHS^ox^ at pH 8.0 and 5 °C in the absence or presence of GdmCl. The initial concentration of R was 10 µM. The folding intermediates were trapped at different reaction times by acidification. (**A**) in the presence of 0.625 M GdmCl; (**B**) in the presence of 0.625 M GdmCl and 5 mM CaCl_2_.

The folding experiments were also performed in the presence of 0.625 M guanidinium chloride (GdmCl) as a denaturant (Figure 9A,B). In the absence of Ca^2+^, I-1 and I-2 were not detected, and a slight amount of N was generated after 10 h. In the presence of Ca^2+^, formation of a significant amount of I-1 was observed, and N yielded 10% after 5 h and 37% after 24 h. The results indicated that I-1 that binds Ca^2+^ keeps the folded conformation even in the presence of 0.625 M GdmCl. On the other hand, I-2 that binds Ca^2+^ would be denatured under the same reaction conditions due to less thermodynamic stability.

## 3. Discussion

Oxidative folding pathways of αLA were reinvestigated herein by using DHS^ox^ as a strong (i.e., with a high oxidation potential) and quantitative oxidant. In the presence of EDTA (under a metal-free condition), no SS rearrangement among the produced SS intermediate ensembles to specific folding intermediates was observed after stoichiometric oxidation of R by DHS^ox^ (see Section 2.2). In the absence of EDTA, on the other hand, one characteristic 3SS intermediate, i.e., I-1, was generated and it was slowly oxidized to N due probably to air oxidation catalyzed by metal ions marginally present in the solution (Figure 3A and Figure 4A). In the presence of Ca^2+^, the oxidative folding progressed more rapidly and the specific 2SS intermediate, i.e., I-2, was additionally generated in an early stage of the folding (Figure 3B and Figure 4B), suggesting that R is transformed to N through I-2 and then I-1. The observed key intermediates, i.e., I-2 and I-1, were then clearly assigned to (61–77, 73–91) with two native SS bonds and des[6–120] with three native SS bonds, respectively, which were previously reported by Chang [35]. They have two common SS bonds, i.e., Cys61-Cys77 and Cys73-Cys91, which are located in the β-sheet domain near the calcium binding site. Thus, only I-2 was a stable intermediate among the 2SS intermediate ensemble in the presence of Ca^2+^. Similarly, only I-1 was a stable intermediate among the 3SS intermediate ensemble. These observations strongly support that complexation of Ca^2+^ to the binding pocket is essential to regeneration of native αLA [35,36].

**Figure 10:**
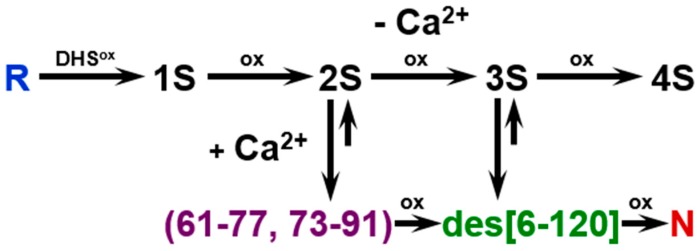
Oxidative folding pathways of αLA by using DHS^ox^. R and N indicate the reduced and native forms of αLA, respectively. 1S, 2S, 3S and 4S are ensembles of intermediates with the indicated number of SS bonds. The SS bonds of the characteristic 2SS intermediate are shown in parentheses and the missing SS bond of the characteristic 3SS intermediate is shown in brackets with a prefix des.

Based on the results discussed above, the oxidative folding pathways of αLA can be delineated as shown in Figure 10. The pathways are in accordance with those previously proposed [7], although slight differences are seen. For example, in previously described pathways, 4SS can be directly converted to N, whereas, in our pathways, 4SS is a dead end. These differences are due to the difference of the folding reagents employed. Our study has also corroborated presence of (61–77, 73–91) and des[6–120] on the pathways to N. By oxidation pulse experiments using DHS^ox^ with high oxidizing ability (Figure 6), it was evidenced that I-1 and I-2 are direct precursors of N. It should be noted that the oxidative folding pathways of αLA would be in sharp contrast to the SS-intact folding scenario: denatured αLA at low pH is well known to form a molten globule, which has the weakly folded α-helix domain and the disordered β-sheet domain [23,24,25,26,27].

In the presence of Na^+^, K^+^, Mg^2+^, and Zn^2+^, which have much smaller association constants to αLA than Ca^2+^, the oxidative folding pathways of αLA were not affected at all (Figure 5). On the other hand, the oxidative folding to N more rapidly proceeded in the presence of Mn^2+^ than in the presence of Ca^2+^. Since SS rearrangement from the SS intermediates to I-1 and I-2 is essential for regeneration of N, it is deducible that I-1 and I-2 are thermodynamically stabilized by binding of Mn^2+^ at the Ca^2+^ binding site. Indeed, it was reported that Mn^2+^ and Ca^2+^ can share the same binding site [46]. The acceleration of the oxidative folding of αLA in the presence of Mn^2+^ may be explained by formation of manganese(II) hydroxide under a basic condition, which would catalyze air oxidation. The effects of temperature and a denaturant (i.e., GdmCl) on the oxidative folding pathways of αLA were also investigated in this study. As a result, it was found that the folding pathways do not change in a range of the applied conditions, while generation of I-1 and I-2 was suppressed at 35 °C and 45 °C and also in the presence of 0.625 M GdmCl. Thermodynamic stability can be ranked in the order of I-2 < I-1 < N. If I-1 and I-2 are not stabilized, αLA must seek N by trial-and-error processes and the folding would result in formation of a large amount of misfolded 4SS intermediates.

Synergetic effects of the Ca^2+^ binding and the Cys61-Cys77 and Cys73-Cys91 disulfide formation during the folding of αLA were frequently discussed in the literature. Wu [51] found that the 2SS variant of αLA having Cys61-Cys77 and Cys73-Cys91 disulfide bonds in the β-sheet domain can bind Ca^2+^, leading to the cooperative formation of substantial tertiary interactions, whereas the other 2SS variant having Cys6-Cys120 and Cys28-Cys111 disulfide bonds in the α-helix domain cannot bind Ca^2+^. A similar Ca^2+^-binding behavior was reported by Hendrix [52], who showed that the 2SS form of αLA with Cys 6-Cys 120 and Cys 28-Cys 111 disulfide bonds being reduced and modified with iodoacetic acid retains the weakly folded β-sheet domain in the presence of Ca^2+^, whereas the α-helix domain is unfolded. The weak Ca^2+^ binding was demonstrated even for a short peptide fragment consisting of residues 72–100 of αLA [53]. These observations are in full agreement with the oxidative folding pathways of αLA shown in Figure 10.

In conclusion, it was corroborated that the oxidative folding pathways of αLA are drastically switched from those like hirudin producing a number of intermediates with statistically formed SS bonds to those like BPTI producing only the (61–77, 73–91) and des[6–120] intermediates by the presence of Ca^2+^, which can stabilize these discrete SS intermediates selectively probably by coordination to the calcium binding site. Similar effects were observed for Mn^2+^ but practically no effect was observed for Na^+^, K^+^, Mg^2+^, and Zn^2+^. Thermodynamic stability increases in the order, (61–77, 73–91) < des[6–120] < N. On the folding pathways (Figure 10), only des[6–120] is stable among four possible des intermediates, i.e., des[6–120], des[28–111], des[61–77] and des[73–91]. This feature is significantly different from the oxidative folding pathways of HEL [15,16,42] although it has the same SS-bonding pattern as αLA (Figure 1). For HEL, three des intermediates, i.e., des[76–94], des[64–80] and des[6–127], can form. Thus, oxidative folding pathways of SS-containing proteins are significantly variable depending on the amino acid sequence and other factors, such as co-existing metal ions, even when the SS-bond topologies are similar to each other.

## 4. Materials and Methods

### 4.1. Materials

Calcium-depleted bovine αLA (L-6010) was obtained from Sigma Aldrich (Tokyo, Japan) and used without purification. Urea and DTT^red^ were obtained from Wako Pure Chemical Industries, Ltd. (Osaka, Japan). GdmCl was obtained from Tokyo Chemical Industry, Ltd. (Tokyo, japan). DHS^ox^ and AEMTS, both of which were not commercially available, were synthesized according to the literature methods [54,55]. Other common chemicals were procured from local companies and were used as obtained.

### 4.2. Preparation of Reduced αLA (R)

Native αLA (6 mg) was dissolved in 600 µL of a 0.1 M tris(hydroxymethyl)aminomethane hydrochloride (Tris) buffer solution at pH 8.0 containing 5 M GdmCl and 0.2 mM DTT^red^. After incubation for 2 h at 25 °C, the solution was passed through a size-exclusion column (Sephadex™ G-25 Fine, Sigma Aldrich, Tokyo, Japan), which was equilibrated at room temperature in a 0.1 M Tris buffer solution at pH 8.0, with a flow rate of 0.8 mL/min. The fraction containing R (4 mL) was collected and was diluted with the same buffer solution to adjust the final concentration of R to be 30 µM. The concentration was determined by the absorbance at 280 nm using a molar extinction coefficient of R (ε_280_ = 27,330 cm^−1^·M^−1^) determined by amino acid analysis and UV spectrometry.

### 4.3. Oxidative Regeneration of Native αLA (N)

The experiments were carried out both in the absence and in the presence of 5 mM CaCl_2_ at pH 8.0 and 5 °C. A solution of R (30 µM, 100 µL) was diluted two-fold with a 0.1 M Tris buffer solution at pH 8.0 or the same buffer solution containing 10 mM CaCl_2_. The obtained solution was manually added with 100 µL of a 30 µM DHS^ox^ solution in a 0.1 M Tris buffer at pH 8.0 containing 0 or 5 mM CaCl_2_. After incubation for 1 min to 24 h, the sample solution was treated with 15 µL of 1 M HCl and then 700 µL of 0.1% trifluoroacetic acid (TFA) in water (acid-quenching) or with 300 µL of 40 mM AEMTS and then 15 µL of 1 M HCl (AEMTS-quenching) to stop the regeneration reaction. The reaction conditions were appropriately modified by changing the pH of the buffer solution (0.1 M Bis-Tris for pH 6.8), the co-existing metal ion (5 mM NaCl, 5 mM KCl, 5 mM MgCl_2_, 5 mM ZnCl_2_, or 0.5 mM MnCl_2_), the reaction temperature (35 or 45 °C), and a denaturant (0.625 M GdmCl) in order to carry out the folding under different conditions.

### 4.4. HPLC Analysis

The sample solutions obtained as described above were analyzed by RP-HPLC. For acid-quenched samples, the solution was directly injected into a ZORBAX 300SBC-18 column (4.6 × 150 mm, 5 µm) (Agilent, Santa Clara, CA, USA) at 25 °C at a flow rate of 0.5 mL/min. Solvent A was 0.1% TFA in water, solvent B was 0.1% trifluoroacetic acid (FTA) in acetonitrile, and a solvent gradient of solvent B (30 to 48% in 60 min) was applied. For AEMTS-quenched samples, the solution was desalted into 0.1 M AcOH through a Sephadex™ G-25 column and then injected into a TSKgel ODS-100V C-18 column (4.6 × 150 mm, 5 µm) (Tosoh, Tokyo, Japan) at 35 °C at a flow rate of 0.5 mL/min. Solvent A was 0.1% TFA in water, solvent B was 0.1% FTA in acetonitrile, and a solvent gradient of solvent B (20 to 37.5% in 10 min and 37.5 to 41.5% in 50 min) was applied. Fractionated folding intermediates were detected by UV at 280 nm.

### 4.5. ESI-MS Analysis

Mass spectra of the fractionated folding intermediates were recorded with the ESI(+) mode on JMS-T100LP mass spectrometer (Jeol, Akishima, Japan). AEMTS-quenched sample solutions were freeze-dried, dissolved in 0.1% formic acid in water and 0.1% formic acid in acetonitrile, and injected through the sample introduction tube. Molecular masses of the analyzed samples were calibrated by using angiotensin I ([M+H]^+^: 1296.685).

## Supplementary Materials

Supplementary materials can be found at www.mdpi.com/1422-0067/18/9/1996/s1.

## Abbreviations

AEMTS	2-Aminoethyl methanethiosulfonate
αLA	Bovine α-lactalbumin
BPTI	Bovine pancreatic trypsin inhibitor
Cys	Cysteine
DHS^ox^	*trans*-3,4-Dihydroxyselenolane oxide
DTT^ox^	*trans*-4,5-Dihydroxy-1,2-dithiane
DTT^red^	Dithiothreitol
EDTA	Ethylenediaminetetraacetic acid
ESI-MS	Electrospray ionization-mass spectrometry
GdmCl	Guanidinium chloride
GSH	Reduced glutathione
GSSG	Oxidized glutathione
HEL	Hen egg white lysozyme
I-1	A folding intermediate of αLA having three native SS bonds but lacking one native SS bond between Cys6-Cys120
I-2	A folding intermediate of αLA having two native SS bonds between Cys61-Cys77 and Cys73-Cys91
N	Native form
R	Fully reduced form
RNase A	Bovine pancreatic ribonuclease A
RP-HPLC	Reverse-phase high performance liquid chromatography
SH	Thiol
SS	Disulfide
TFA	Trifluoroacetic acid
Tris	Tris(hydroxymethyl)aminomethane hydrochloride
