# Supplementary material for: Effects of Metal Ions, Temperature, and a Denaturant on the Oxidative Folding Pathways of Bovine α-Lactalbumin
Source: Int J Mol Sci. 2017 Sep 16;18(9):1996. doi: 10.3390/ijms18091996 (PMC5618645; doi:10.3390/ijms18091996)
Supplement: Supplementary file 1 [file ijms-18-01996-s001.pdf]

International Journal of
Molecular Sciences

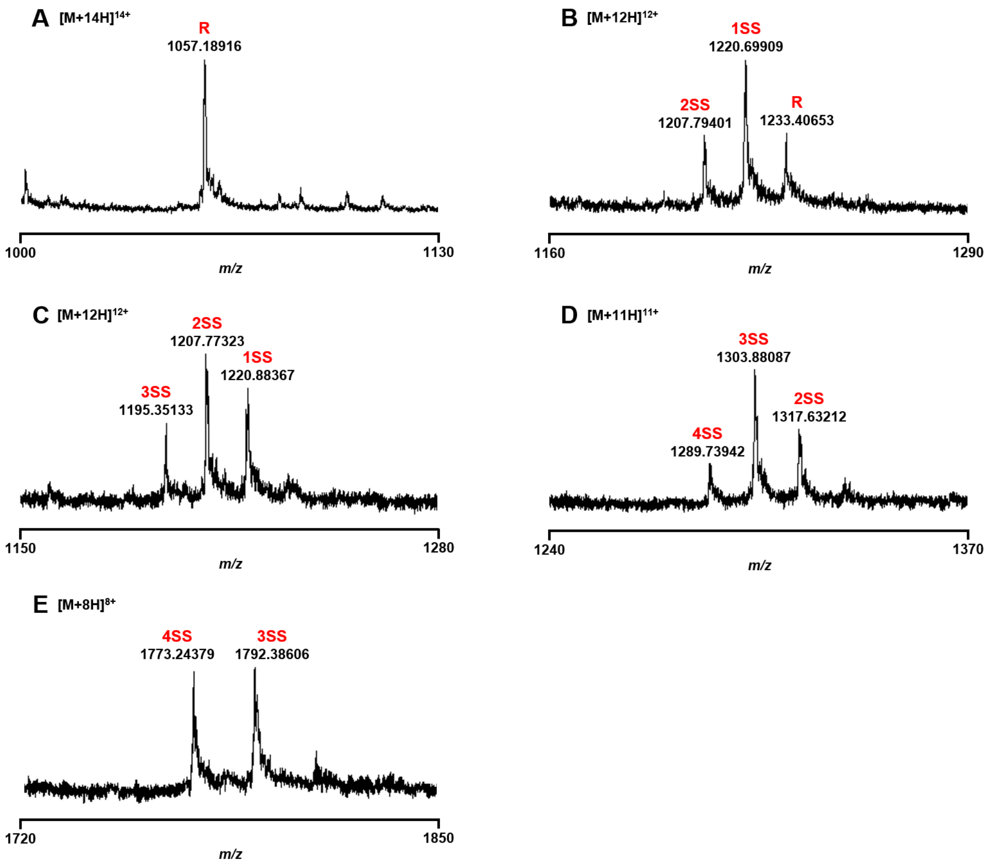

Figure S1. ESI mass spectra of the SS intermediates generated by the reaction of αLA with DHS^{ox}. The reaction was carried out in Tris-HCl buffer (pH 8.0) containing 2 mM EDTA. After 1 min, the reaction was quenched by AEMTS. The samples obtained were analyzed with the ESI(+) mode. (**A**) R; (**B**) 1 eq DHS^{ox} was added to R; (**C**) 2 eq DHS^{ox} was added to R; (**D**) 3 eq DHS^{ox} was added to R; (**E**) 4 eq DHS^{ox} was added to R.

Table S1. Assignment of the peaks observed in Figure S1. [a]

Species	Expected M+	$[M+8H]^{8+}$	$[M+11H]^{11+}$	$[M+12H]^{12+}$	$[M+14H]^{14+}$
R + 8×AEMTS	14784.9862			1233.40653<br>(1233.0899)	1057.18916<br>(1057.0782)
1SS + 6×AEMTS	14632.9420			1220.69909<br>1220.88367<br>(1220.4196)	
2SS + 4×AEMTS	14480.8978		1317.63212<br>(1317.4530)	1207.79401<br>1207.77323<br>(1207.7492)	
3SS + 2×AEMTS	14328.8536	1792.38606<br>(1792.1144)	1303.88087<br>(1303.6308)	1195.35133<br>(1195.0789)	
4SS	14176.8094	1773.24379<br>(1773.1089)	1289.73942<br>(1289.8086)		

[a] The calculated mass numbers are shown in parentheses.

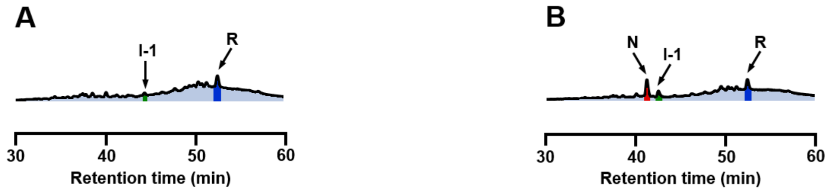

Figure S2. RP-HPLC chromatograms obtained by the oxidation of R (10 μM) with 1 eq DHS^{ox} at pH 6.8 and 5 °C in the absence of EDTA. The reaction was acid-quenched after 20 h. For HPLC analysis conditions, see the experimental section. (**A**) In the absence of $CaCl_2$; (**B**) In the presence of 5 mM $CaCl_2$.

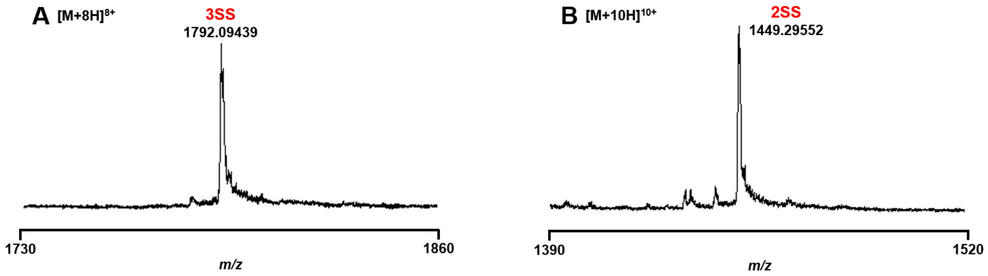

Figure S3. ESI mass spectra of I-1 and I-2 quenched by AEMTS. (**A**) I-1; (**B**) I-2.

Table S2. Assignment of the peaks observed in Figure S3. [a]

	Expected M^+	$[M+8H]^{8+}$	$[M+10H]^{10+}$
I-1 (3SS) + 2×AEMTS	14328.8536	1792.09439 (1792.1144)	
I-2 (2SS) + 4×AEMTS	14480.8978		1449.29552 (1449.0975)

[a] The calculated mass numbers are shown in parentheses.

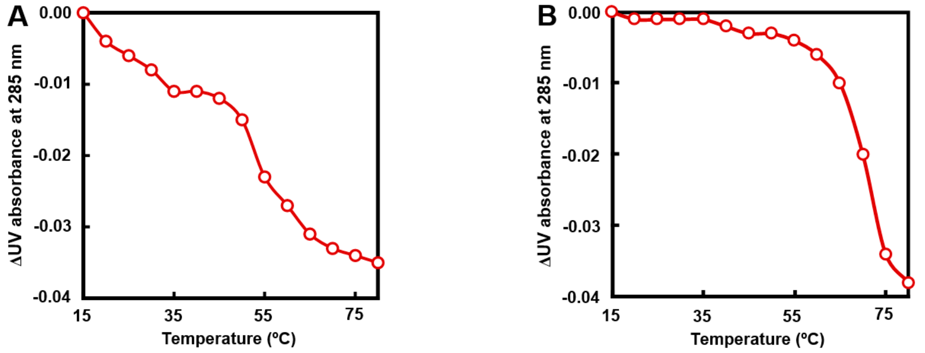

Figure S4. The thermal denaturation curves of αLA (10 μM) in the absence (**A**) and presence (**B**) of 5 mM $CaCl_2$.
